# Diagnostic Performance of Parotid Shear-Wave Elastography for Predicting Histopathological Positivity in Patients with Suspected Primary Sjögren’s Syndrome

**DOI:** 10.3390/diagnostics16071095

**Published:** 2026-04-05

**Authors:** Ozlem Unal, Betul Akdal Dolek, Ahmet Kor, Eda Sener Alcın, Sukran Erten

**Affiliations:** 1 Department of Radiology, Bilkent City Hospital, Ankara Yıldırım Beyazıt University, 06800 Ankara, Turkey; 2 Department of Radiology, Bilkent City Hospital, 06800 Ankara, Turkey; 3 Department of Rheumatology, Aksaray University Education and Research Hospital, 68100 Aksaray, Turkey; 4 Department of Rheumatology, Bilkent City Hospital, Ankara Yıldırım Beyazıt University, 06800 Ankara, Turkey

**Keywords:** Sjögren’s syndrome, parotid gland, elastography, shear-wave elastography, biopsy, ROC analysis

## Abstract

**Background:** Primary Sjögren’s syndrome (pSS) is a chronic autoimmune epithelitis characterized by lymphocytic infiltration of the exocrine glands. Although labial salivary gland biopsy remains the reference standard for diagnosis, it is invasive and may not always be feasible in routine practice. This study aimed to evaluate the diagnostic performance of parotid gland shear-wave elastography (SWE) and to investigate its relationship with histopathological findings in patients with suspected pSS. **Methods:** This prospective study included 93 participants (53 patients with pSS and 40 controls). Shear-wave elastography measurements of the parotid glands were obtained, and their association with histopathological findings was analyzed. Diagnostic performance was assessed using receiver operating characteristic (ROC) analysis. Multivariable logistic regression was performed to evaluate independent predictors of histopathological positivity. **Results:** Mean shear-wave elastography velocity values (m/s) were significantly higher in the pSS group than in controls (*p* < 0.001), and this difference remained significant after adjustment for age (adjusted β = 2.141, *p* < 0.001). ROC analysis demonstrated moderate discriminative performance for predicting histopathological positivity (AUC = 0.76, 95% CI: 0.61–0.89). The optimal cut-off value of 2.17 m/s yielded a sensitivity of 69.0% and a specificity of 94.1%. A moderate positive correlation was observed between right parotid elastography values and histopathological grade (r = 0.483, *p* < 0.001). In multivariable analysis, elastography mean and anti-SSA positivity showed positive but non-significant associations with histopathological positivity. The model demonstrated good calibration (Hosmer–Lemeshow *p* = 0.866) and high apparent discrimination (AUC = 0.947), with reduced performance after internal validation. **Conclusions:** Parotid shear-wave elastography is a non-invasive imaging method with moderate diagnostic performance in pSS. Elastography measurements correlate with histopathological involvement and remain significantly elevated after age adjustment. SWE may serve as a complementary tool for pre-biopsy risk stratification, particularly when biopsy is contraindicated or declined. Further validation in larger, independent cohorts is required.

## 1. Introduction

Primary Sjögren’s syndrome (pSS) is a chronic, systemic autoimmune epithelitis characterized by progressive lymphocytic infiltration of the exocrine glands, most prominently the salivary and lacrimal glands [1]. The disease represents one of the most frequently encountered connective tissue disorders in rheumatology practice, with an estimated incidence of 6.0–11.8 per 100,000 person-years in Asia and 3.9–5.3 in Europe [1,2]. pSS predominantly affects middle-aged women, with a female-to-male ratio of approximately 9:1 [1,3]. Clinically, the disease most commonly presents with the sicca complex characterized by xerostomia (dry mouth) and xerophthalmia (dry eyes). However, approximately 30–40% of patients may develop significant extraglandular manifestations involving organs such as the lungs, kidneys, joints, and nervous system [1,3]. Patients with pSS carry a markedly increased risk of malignant lymphoma compared with the general population [1].

Despite advances in diagnostic approaches, the diagnosis of pSS remains challenging due to the clinical heterogeneity of the disease and the non-specific nature of its presenting symptoms [1]. According to the 2016 ACR/EULAR classification criteria, anti-SSA/Ro antibody positivity and labial salivary gland biopsy constitute the most heavily weighted objective criteria for classification [1,3]. Histopathological evidence of focal lymphocytic sialadenitis with a focus score ≥ 1 in labial salivary gland biopsy specimens is widely regarded as the diagnostic reference standard. Nevertheless, biopsy is associated with several disadvantages, including its invasive nature, potential patient discomfort, and the possibility of complications such as persistent sensory disturbances [2,3]. In addition, the diagnostic yield may vary depending on biopsy sampling adequacy and the experience of the evaluating pathologist [1,2].

Historically, several conventional methods have been used to assess salivary gland function and structure, including sialometry, parotid sialography, and salivary gland scintigraphy. However, the clinical utility of these techniques has gradually declined due to their inherent limitations. Sialography is invasive and frequently uncomfortable for patients, whereas scintigraphy requires exposure to ionizing radiation and both techniques demonstrate relatively limited specificity [4,5]. Consequently, interest has increased in non-invasive, repeatable, and cost-effective imaging techniques that may assist in pre-biopsy risk stratification in patients with suspected pSS [2,3].

B-mode ultrasonography (US) has emerged as an important imaging modality for evaluating structural alterations and parenchymal heterogeneity in the salivary glands and has demonstrated meaningful diagnostic value in primary Sjögren’s syndrome, with previous studies reporting significant correlations between ultrasonographic abnormalities and clinical parameters [5,6,7]. Despite its widespread use in clinical practice, the technique remains operator-dependent and largely semi-quantitative, which represents an important limitation for routine diagnostic evaluation [5,6]. In recent years, ultrasound elastography (USE), particularly shear-wave elastography (SWE), has gained increasing attention as a promising imaging technique by enabling quantitative measurement of tissue stiffness [2,3,4]. In pSS, glandular stiffness increases due to lymphocytic infiltration, ductal epithelial hyperplasia, and progressive fibrosis, providing a biological basis for elastographic measurements [3,4]. A recent systematic review and meta-analysis demonstrated that shear-wave elastography provides moderate overall diagnostic accuracy for the detection of primary Sjögren’s syndrome [8].

According to recent meta-analyses, elastography demonstrates relatively high sensitivity and specificity in distinguishing patients with pSS from non-pSS individuals [8]. However, direct evidence comparing quantitative elastography measurements with histopathological reference standards and defining clinically useful thresholds for predicting biopsy positivity remains limited. Although recent studies have highlighted the increasing role of salivary gland imaging in the diagnostic work-up of pSS, the potential contribution of quantitative elastography for guiding biopsy decision-making has not yet been clearly defined. Evaluating the association between elastography-derived stiffness measurements and histopathological findings may provide further insight into the potential role of elastography in diagnostic algorithms for primary Sjögren’s syndrome.

The association between quantitative parotid elastography measurements and histopathological findings was evaluated in patients with suspected primary Sjögren’s syndrome. The primary aim was to evaluate the diagnostic performance of shear-wave elastography in predicting histopathological positivity. As a secondary objective, we evaluated whether elastography parameters could predict biopsy positivity and identify a clinically relevant stiffness cut-off value.

## 2. Materials and Methods

### 2.1. Study Design and Setting

This prospective, observational diagnostic accuracy study was conducted at Ankara Bilkent City Hospital. The study design followed the STARD (Standards for Reporting Diagnostic Accuracy Studies) recommendations for diagnostic accuracy research. Consecutive adult patients (≥18 years) evaluated for suspected primary Sjögren’s syndrome (pSS) according to the 2016 ACR/EULAR classification criteria [1,3] and referred for labial salivary gland biopsy for diagnostic confirmation were included. Because previous studies have not specifically evaluated the ability of parotid shear-wave elastography to predict histopathological positivity, reliable effect size estimates required for formal sample size calculation were not available. Therefore, the sample size was determined by the number of consecutive eligible patients evaluated during the study period.

### 2.2. Participants

Patients were recruited consecutively from individuals referred for evaluation of suspected pSS. Inclusion criteria were age ≥ 18 years and clinical suspicion of pSS according to the 2016 ACR/EULAR classification criteria [1,3].

All participants were evaluated during the diagnostic work-up for suspected primary Sjögren’s syndrome.

Exclusion criteria included a history of head and neck radiotherapy, prior parotid or submandibular gland surgery, active hepatitis C infection, HIV infection, sarcoidosis, or IgG4-related disease.

#### 2.2.1. Control Group

The control group consisted of individuals presenting with sicca symptoms who were positive for antinuclear antibodies (ANA) but negative for the extractable nuclear antigen (ENA) profile (anti-SSA and anti-SSB). Histopathological examination of labial salivary gland biopsy samples in these individuals demonstrated normal findings with a focus score < 1. These patients were included as controls because they represent a frequent clinical situation in which individuals with sicca symptoms and positive ANA are evaluated for suspected primary Sjögren’s syndrome but do not meet histopathological or serological criteria for the disease.

#### 2.2.2. Patient Group

The patient group consisted of individuals presenting with sicca symptoms together with anti-SSA and/or anti-SSB positivity and abnormal findings in objective tests, including Schirmer test ≤ 5 mm/5 min or unstimulated salivary flow rate ≤ 0.1 mL/min. These patients fulfilled the 2016 ACR/EULAR classification criteria for primary Sjögren’s syndrome.

### 2.3. Ultrasonographic Assessment

Shear-wave elastography (SWE) examinations were performed using a LOGIQ E8 ultrasound system (GE Healthcare, Chicago, IL, USA). All measurements were obtained using a high-frequency 9L linear probe operating at 6–15 MHz.

Shear-wave velocity (m/s) was preferred over Young’s modulus (kPa), as it represents a directly measured parameter and is less influenced by system-specific conversion assumptions, allowing more consistent comparisons across studies, particularly in structurally heterogeneous tissues such as salivary glands in pSS. This approach is also consistent with previously published studies.

Patients were examined in the supine position with slight neck extension, and both parotid glands were evaluated bilaterally.

During elastography assessment, a standardized region of interest (ROI) approximately 1 cm^2^ in size was positioned within the glandular parenchyma. Large vessels and ductal structures were excluded from the measurement area. For each gland, at least three consecutive measurements were obtained, and mean values were used in subsequent statistical analyses.

Probe compression was carefully minimized during elastography acquisition, and measurements were obtained during breath-hold to reduce motion artifacts.

All examinations were performed using a standardized acquisition protocol, and the mean value of repeated ROI measurements was used for analysis to reduce measurement variability. This protocol was consistent with previously proposed recommendations for the standardization of salivary gland ultrasonography in Sjögren’s syndrome. All measurements were performed using a single ultrasound system to ensure internal consistency.

Primary SWE examinations were performed by a single radiologist with more than 10 years of experience in head and neck ultrasonography, who was blinded to the clinical, serological, and histopathological data of the participants. For interobserver reliability analysis, a second radiologist with more than 8 years of experience independently performed SWE measurements in all study participants using the same standardized acquisition protocol. Interobserver agreement between the two observers was assessed using the intraclass correlation coefficient (ICC) based on a two-way random-effects model with absolute agreement. ICC values were interpreted as follows: <0.50 was considered poor, 0.50–0.75 moderate, 0.75–0.90 good, and >0.90 excellent reliability. Intraobserver variability was not formally assessed.

### 2.4. Reference Standard: Histopathological Evaluation

All participants underwent labial salivary gland biopsy for diagnostic confirmation. Shear-wave elastography and labial salivary gland biopsy were performed within the same diagnostic evaluation period, without a clinically meaningful time interval between the two assessments.

Biopsies were performed using the standard technique. Histopathological evaluation was conducted according to established criteria. The presence of ≥1 lymphocytic focus per 4 mm^2^ of glandular tissue was defined as a positive biopsy, corresponding to focal lymphocytic sialadenitis.

### 2.5. Statistical Analysis

Statistical analyses were performed using appropriate statistical software. Continuous variables were presented as mean ± standard deviation or median with interquartile range (IQR), while categorical variables were expressed as counts and percentages.

Normality of distribution was assessed using the Shapiro–Wilk test.

Group comparisons were performed using Student’s *t*-test or Mann–Whitney U test depending on distribution characteristics. Categorical variables were analyzed using the chi-square test.

The relationship between elastography measurements and histopathological findings was evaluated using Pearson or Spearman correlation analysis where appropriate.

Diagnostic performance was assessed using receiver operating characteristic (ROC) curve analysis. The area under the ROC curve (AUC), sensitivity, specificity, and optimal cut-off values were calculated using the Youden index. The cut-off value reported in Section 3 corresponds to the threshold obtained from the Youden index.

To identify independent predictors of biopsy positivity, multivariable logistic regression analysis was performed. Model calibration was assessed using the Hosmer–Lemeshow goodness-of-fit test, while model discrimination was evaluated using ROC analysis.

Internal validation of the multivariable logistic regression model was conducted using bootstrap resampling with 1000 iterations to evaluate model optimism and obtain a corrected AUC. The events-per-variable ratio was considered acceptable given the exploratory nature of the model, although still relatively limited.

A formal post hoc power calculation was not performed. Considering the exploratory design of the study and the limited availability of prior data for effect size estimation, the sample size was based on consecutive patient inclusion during the study period. This should be taken into account when interpreting the results of the multivariable analysis.

As a sensitivity analysis, nearest-neighbor age matching was performed.

A *p*-value < 0.05 was considered statistically significant.

### 2.6. Ethical Considerations

The study was conducted in accordance with the principles of the Declaration of Helsinki. Written informed consent was obtained from all participants prior to inclusion in the study.

A total of 93 individuals were included in the analysis. The relationship between non-invasive elastography parameters and histopathological biopsy results was evaluated.

## 3. Results

**Figure 1:**
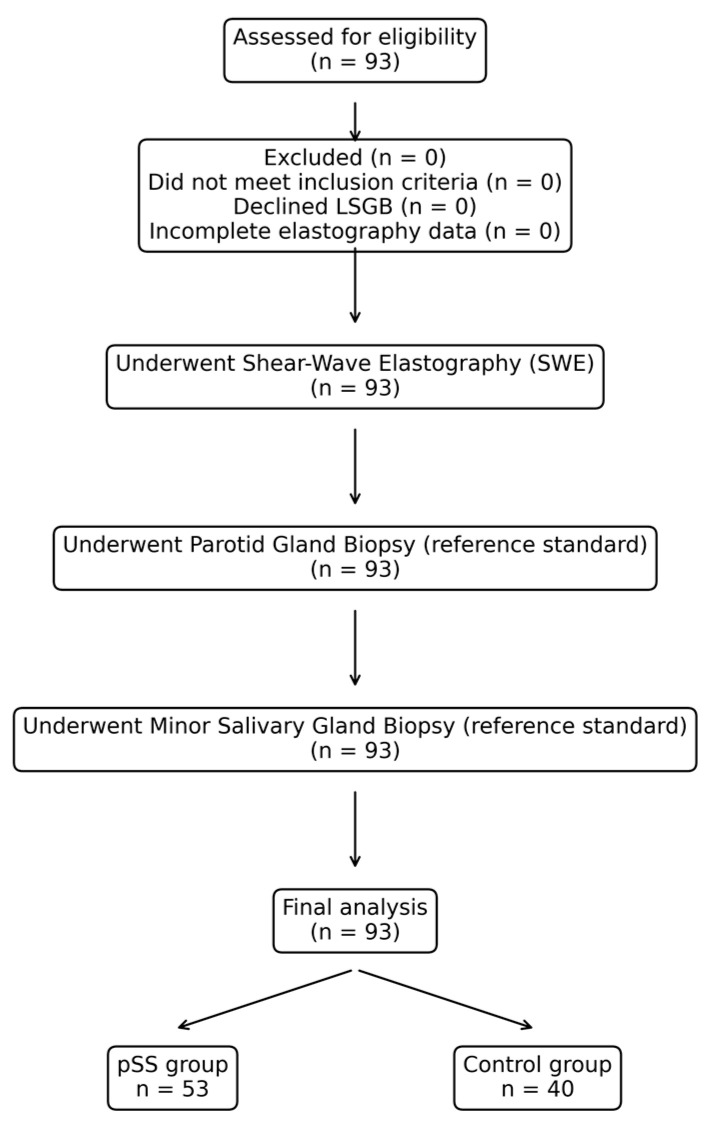
STARD-compliant flow diagram of participant selection, index testing (SWE), and reference standard assessment (labial salivary gland biopsy).

The participant flow according to STARD recommendations is presented in Figure 1. All eligible individuals underwent both the index test (shear-wave elastography) and the reference standard (labial salivary gland biopsy), and no participants were excluded from the final analysis.

A total of 93 individuals were included in the study, comprising 53 patients in the pSS group and 40 subjects in the control group. The mean age of the study population was 48.7 ± 12.9 years. All participants were female; therefore, no between-group comparison was performed for sex. The baseline demographic and clinical characteristics of the study population are summarized in Table 1.

Elastography measurements were obtained by a single experienced radiologist using multiple ROI acquisitions per gland, and the mean values were used for analysis.

A statistically significant difference in mean elastography shear-wave velocity values (m/s) was observed between the patient and control groups (*p* < 0.001). Patients with Sjögren’s syndrome demonstrated significantly higher shear-wave elastography velocity values (m/s) than controls, indicating increased salivary gland stiffness (Table 2).

Interobserver reliability analysis demonstrated moderate to good agreement between the two observers. The intraclass correlation coefficient (ICC) was 0.735 for the right parotid gland and 0.787 for the left parotid gland (Table 3).

After adjustment for age using ANCOVA, shear-wave elastography velocity values (m/s) remained significantly higher in the pSS group (adjusted β = 2.141, 95% CI: 1.468–2.814; *p* < 0.001) (Table 4). In an additional age-matched analysis, shear-wave elastography velocity values (m/s) remained significantly higher in the pSS group (Supplementary Table S1).

According to the Shapiro–Wilk test, age was normally distributed, whereas shear-wave elastography velocity parameters (m/s) showed a non-normal distribution. Therefore, parametric or non-parametric tests were applied as appropriate. Shear-wave elastography velocity measurements (m/s) also differed significantly across ultrasonographic and histopathological grades (*p* < 0.05).

**Figure 2:**
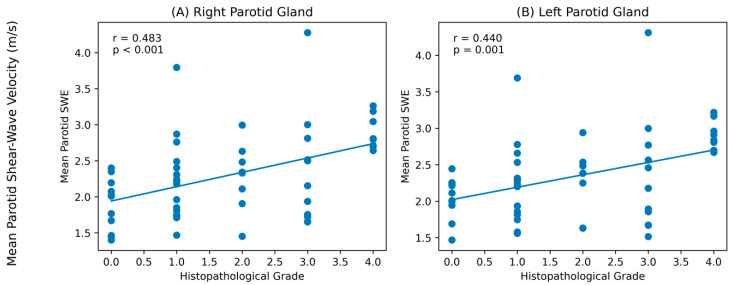
Correlation between mean parotid shear-wave elastography velocity (m/s) and histopathological grade. (**A**) Right parotid gland. (**B**) Left parotid gland. Dots represent individual data points, and the solid line indicates the fitted regression line.

Correlation analysis using Spearman’s test demonstrated a moderate positive correlation between the mean right parotid gland shear-wave elastography velocity (m/s) and pathological grade (r = 0.483, *p* < 0.001). A weaker but still significant positive correlation was also observed for the mean left gland shear-wave elastography velocity (m/s) (r = 0.440, *p* = 0.001) (Figure 2).

**Figure 3:**
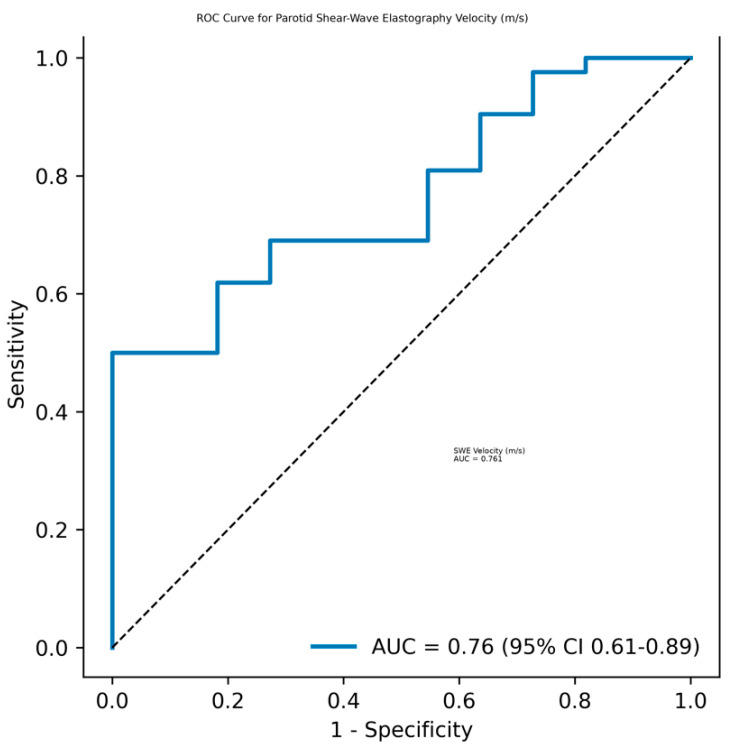
Receiver operating characteristic (ROC) curve demonstrating the diagnostic performance of parotid shear-wave elastography velocity (m/s) for predicting histopathological positivity. The dashed line indicates the line of no discrimination (AUC = 0.5).

Receiver operating characteristic (ROC) analysis demonstrated moderate discriminative performance of elastography parameters (AUC = 0.76, 95% CI: 0.61–0.89) (Figure 3). The optimal shear-wave elastography velocity cut-off value (m/s), determined using the Youden index, was 2.17, yielding a sensitivity of 69.0% and a specificity of 94.1%. The diagnostic performance of this threshold is summarized in Table 5. The relatively high specificity observed at this threshold suggests that shear-wave elastography may be useful as a rule-in diagnostic tool, particularly in selected clinical contexts.

**Figure 4:**
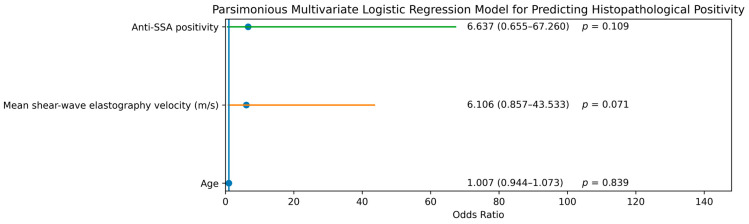
Forest plot of the parsimonious multivariate logistic regression model evaluating predictors of histopathological positivity. The model included age, mean shear-wave elastography velocity (m/s), and anti-SSA positivity. Dots represent odds ratios (ORs), and horizontal lines indicate 95% confidence intervals (CIs). Different colors are used to distinguish variables.

In the parsimonious multivariate logistic regression analysis including age, mean shear-wave elastography velocity (m/s), and anti-SSA positivity, none of the variables reached independent statistical significance for predicting histopathological positivity. Mean shear-wave elastography velocity (m/s) and anti-SSA positivity showed non-significant trends toward association, likely reflecting the limited sample size (Table 6, Figure 4).

Although relatively large effect sizes were observed for elastography velocity, these findings should be interpreted with caution, as they likely reflect the small sample size and limited number of events per variable, which may have reduced the precision of the estimates.

The overall logistic regression model demonstrated good calibration (Hosmer–Lemeshow *p* = 0.866). The apparent discriminative performance of the model was high (AUC = 0.947). This high apparent performance should be interpreted with caution, as it likely reflects overfitting related to the limited sample size and number of events. Bootstrap internal validation revealed a degree of optimism, and the optimism-corrected AUC decreased to 0.72. This reduction suggests that the initial model performance was likely overestimated, and the corrected AUC more accurately reflects the model’s true discriminative ability. This corrected value is comparable to the diagnostic performance observed in the primary ROC analysis of elastography measurements.

**Figure 5:**
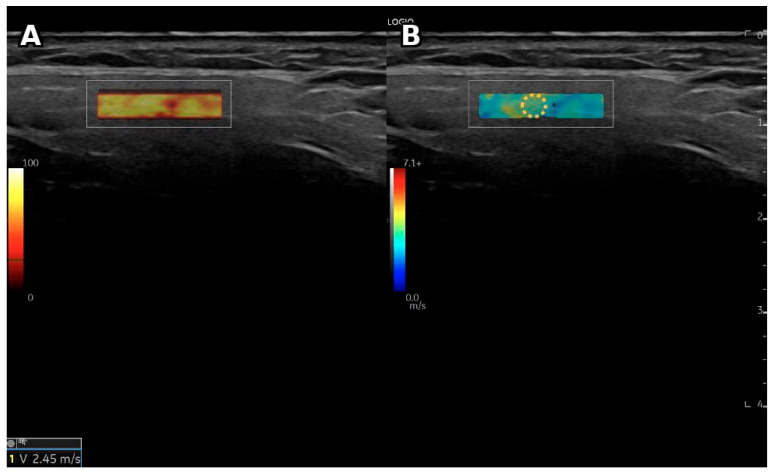
Representative B-mode ultrasonography and shear-wave elastography (SWE) images of the parotid gland in a patient with primary Sjögren’s syndrome. (**A**) B-mode ultrasonography showing heterogeneous parenchymal echotexture of the parotid gland. (**B**) Corresponding SWE velocity map demonstrating increased tissue stiffness within the region of interest (ROI). The dotted circle indicates the region of interest (ROI). Warmer colors indicate higher shear-wave velocity values (m/s).

A representative ultrasound and shear-wave elastography image of the parotid gland obtained from a patient with primary Sjögren’s syndrome is presented in Figure 5.

## 4. Discussion

In this study, salivary gland stiffness measured by shear-wave elastography was significantly higher in patients with primary Sjögren’s syndrome than in symptomatic controls. This increase in stiffness is consistent with the known pathophysiological processes of pSS, including lymphocytic infiltration, ductal epithelial proliferation, and progressive fibrosis, all of which alter the mechanical properties of salivary gland tissue. Only a few prospective studies have evaluated the relationship between quantitative parotid elastography measurements and histopathological findings in patients with suspected pSS. In this study, shear-wave velocity was reported as the primary elastography parameter, as it reflects the directly measured property and may be less affected by assumptions related to tissue composition [3,4].

In this cohort, ROC analysis demonstrated that elastography had moderate diagnostic performance for distinguishing patients with pSS. The observed AUC was slightly lower than that reported by Satış et al., but remained within the range described in previous studies. Recent meta-analyses have reported moderate but heterogeneous diagnostic accuracy of salivary gland elastography in pSS [8]. These findings suggest that elastography should be interpreted alongside clinical and imaging findings rather than used in isolation. In this context, previous studies have also indicated that elastography may provide additional diagnostic information when combined with conventional ultrasonography-based models including in salivary gland tumors [9], which supports the broader applicability of elastography as a complementary imaging tool.

In the present study, the cut-off value of 2.17 m/s identified using the Youden index showed high specificity and acceptable sensitivity. This suggests that elastography may be useful as a rule-in tool in selected clinical contexts. In practice, this may help identify patients who are more likely to benefit from biopsy, potentially reducing unnecessary invasive procedures. When compared with previous studies, the proposed threshold falls within a similar range, although some variability exists. Several studies, including Arslan et al., have reported parotid shear-wave elastography cut-off values between approximately 1.9 and 2.5 m/s, depending on the study population and measurement approach [3,10]. This variability likely reflects differences in patient characteristics, disease spectrum, ultrasound systems, acquisition protocols, and operator experience. It should be considered when interpreting the proposed threshold, as measurements may vary across ultrasound systems. Therefore, the cut-off value identified in this study should be interpreted within the context of the applied methodology and should not be considered directly generalizable. External validation in independent cohorts, preferably using standardized acquisition protocols, will be essential before this threshold can be reliably applied in routine clinical practice.

Another important aspect of this study is the evaluation of histopathological correlations. We observed a significant positive association between mean shear-wave elastography velocity values and biopsy-based pathological grading in both parotid glands, with a slightly stronger association in the right gland. Previous studies, including that by Mo et al., have reported similar relationships between ultrasound findings and histopathological focus scores. These findings suggest that elastography may reflect the degree of glandular involvement rather than serving solely as a structural imaging tool. In our study, histopathological confirmation was based on labial salivary gland biopsy, which remains the reference standard according to the ACR/EULAR classification criteria.

The selection of ANA-positive symptomatic individuals as controls reflects a clinically relevant population encountered in routine practice. However, this approach may introduce spectrum bias, as these individuals may represent a heterogeneous or intermediate group rather than truly healthy controls. This should be considered when interpreting the diagnostic performance of elastography in this study. These findings should also be interpreted in the context of a population in which most patients had not yet initiated disease-specific therapy. Future studies that include both healthy controls and disease controls would help to further clarify this issue.

In the parsimonious multivariable model, age, mean elastography velocity, and anti-SSA positivity were associated with histopathological positivity; however, none reached statistical significance. Although relatively large effect sizes were observed for elastography velocity together with a borderline *p*-value, this likely reflects the small sample size and limited number of events per variable, which may have reduced the precision of the estimates. These findings should be interpreted with caution and do not support firm conclusions regarding independent predictive value and should therefore be considered exploratory. The lack of statistical significance in the multivariable analysis may be related to limited statistical power.

Despite this, the model demonstrated high apparent discriminative performance. However, this should be interpreted with caution. High apparent AUC values in small datasets may reflect overfitting rather than true predictive ability. After bootstrap correction, the decrease in AUC indicated a degree of model optimism, which is not unexpected in studies with relatively small sample sizes.

The matrix risk model proposed by Mo et al. suggested that elevated elastography values may predict biopsy positivity. Our findings are consistent with this interpretation and support the potential role of elastography in pre-biopsy risk assessment. In clinical practice, elastography may help reduce diagnostic uncertainty, particularly when biopsy is contraindicated or declined by the patient. The persistence of significant differences after age adjustment suggests that increased gland stiffness is not solely attributable to age-related changes.

Shear-wave elastography may therefore provide complementary information during the diagnostic evaluation of primary Sjögren’s syndrome. When interpreted together with clinical findings, serological markers, and conventional ultrasonographic features, elastography measurements may improve risk stratification and help guide decisions regarding gland biopsy. This is in line with previous studies demonstrating the significant diagnostic contribution of salivary gland ultrasonography within the ACR/EULAR classification framework [11]. However, elastography should be regarded as an adjunct imaging biomarker rather than a substitute for biopsy.

Another potential advantage of SWE is its contribution to the standardization of imaging assessment in pSS. Quantitative elastography parameters may complement grayscale ultrasonography by providing objective measurements of tissue stiffness. This combined approach may help identify patients more likely to have positive biopsy findings and support more selective use of invasive procedures.

Probe-related pre-compression may influence elastography measurements, particularly in superficially located structures such as the parotid gland. Although efforts were made to minimize probe pressure, some degree of compression is unavoidable. The use of adjunct techniques such as ultrasound gel pads may help reduce pressure-related variability and could be considered in future studies.

Standardization of salivary gland ultrasonography protocols is essential to improve reproducibility and reliability of ultrasound-based assessments. Consistent acquisition protocols and appropriate operator training remain critical for obtaining reliable elastography measurements.

The moderate to good interobserver agreement observed in this study supports the reproducibility of shear-wave elastography measurements. The standardized acquisition protocol may have contributed to reducing operator-dependent variability.

The moderate AUC observed in our cohort likely reflects both the heterogeneous pattern of glandular involvement in pSS and the limited discriminative ability of elastography when used in isolation. Nevertheless, the relatively high specificity at the selected cut-off suggests that elastography may be useful as a rule-in tool in selected clinical contexts, although these findings should be interpreted with caution. Future multiparametric approaches that combine SWE parameters with ultrasonographic scores, serological markers, and clinical variables may further improve diagnostic accuracy.

Overall, our findings indicate that shear-wave elastography provides additional information in patients with suspected pSS. The observed association between elastography measurements and histopathological findings supports the potential role of quantitative imaging biomarkers in autoimmune glandular diseases. However, several factors should be taken into account when interpreting these results. The study was conducted in a single center and included only female participants, which may limit generalizability. In clinical practice, non-invasive imaging techniques such as SWE may help refine patient selection for biopsy rather than replace it. At the same time, these findings should not be considered sufficient for clinical decision-making without external validation. Further prospective multicenter studies are needed to better define the role of elastography within the diagnostic pathway of primary Sjögren’s syndrome.

This study has several important strengths. First, quantitative shear-wave elastography measurements were directly compared with histopathological findings obtained from labial salivary gland biopsy, which remains the reference standard for diagnosing primary Sjögren’s syndrome. Combining imaging data with biopsy-based pathological grading allowed a clearer assessment of the relationship between glandular stiffness and histopathological disease severity.

Second, elastography measurements were systematically obtained from both parotid glands using multiple region-of-interest acquisitions. This approach improved the reliability of stiffness measurements and helped reduce measurement variability.

Third, several complementary statistical methods were applied, including correlation analysis, receiver operating characteristic (ROC) analysis, and multivariable logistic regression modeling. These analyses provided a broader evaluation of the diagnostic and predictive relevance of elastography.

Finally, the inclusion of a symptomatic control group with clinical suspicion of pSS allowed assessment of elastography performance in a clinically relevant population that closely reflects routine diagnostic practice.

This study also has several limitations. First, it was conducted at a single center, which may limit the generalizability of the findings. The study population also consisted entirely of female participants. Although this reflects the known female predominance in primary Sjögren’s syndrome, it may limit extrapolation of the results to male patients. Moreover, single-center studies may be influenced by local referral patterns, patient selection, and imaging practices, which could affect the observed diagnostic performance and limit generalizability to other clinical settings.

Second, despite the expanded sample size, the events-per-variable ratio remained relatively limited for multivariable modeling. The sample size was also relatively small for multivariable analysis, which may have reduced the ability to detect independent associations; therefore, non-significant findings should be interpreted with caution. Other salivary gland conditions such as tumors or IgG4-related disease were not systematically evaluated in this cohort. Information on disease duration and treatment status was limited, and subgroup analyses based on treatment exposure were not performed. As patients were evaluated during the diagnostic work-up phase, most had not yet initiated disease-specific therapy.

Although interobserver reliability analysis demonstrated moderate to good agreement, all elastography examinations were performed by experienced radiologists at a single institution. This may limit the broader generalizability of the findings.

Most shear-wave elastography measurements were also obtained by a single experienced radiologist. Despite the use of a standardized acquisition protocol, some degree of operator-related variability cannot be excluded. Probe-related pre-compression may have influenced elastography measurements despite efforts to minimize pressure.

In addition, elastography measurements may vary across different ultrasound systems due to differences in hardware and processing algorithms. Therefore, the cut-off values reported in this study may be system-dependent and may not be directly transferable to devices from other manufacturers without external validation. Future multicenter studies evaluating elastography across different ultrasound systems are required to confirm the generalizability of the proposed cut-off values.

Intraobserver variability was not evaluated, which may represent a potential source of measurement variability.

Finally, the cross-sectional design does not allow assessment of temporal relationships between elastography measurements and disease progression.

Future multicenter prospective studies will be needed to clarify the role of SWE in the diagnostic pathway of pSS. In particular, studies including larger and more heterogeneous populations, different operators, and varied ultrasound systems will be essential to confirm the robustness and reproducibility of these findings across clinical settings. Prospective validation of standardized threshold values and evaluation of their potential incorporation into ACR/EULAR classification criteria will also be important.

Our findings suggest that quantitative elastography may be incorporated into the routine ultrasonographic evaluation of patients with suspected pSS. The consistent relationship between elastography measurements and histopathological findings observed in both parotid glands supports the potential role of SWE as a quantitative imaging biomarker reflecting glandular involvement.

Although independent predictive significance was not demonstrated in the multivariable model, the relatively large effect size observed for mean shear-wave elastography velocity, together with the high discriminative performance of the model, suggests that combining elastography parameters with anti-SSA positivity may support integrated risk stratification, although this requires further validation. This approach may improve diagnostic confidence in borderline cases.

## 5. Conclusions

This study shows that shear-wave elastography is a non-invasive imaging technique with moderate diagnostic accuracy for assessing salivary gland involvement in primary Sjögren’s syndrome. Elastography velocity values were significantly higher in patients with pSS and were consistently associated with histopathological findings.

The persistence of higher elastography values after age adjustment suggests that these differences are unlikely to be explained solely by age-related changes. The consistent findings across both glands support the idea that elastography reflects underlying disease-related changes in glandular stiffness.

Although independent predictive significance was not demonstrated in the multivariable analysis, elastography may still provide useful complementary information in clinical practice, particularly when considering the need for biopsy.

In this context, shear-wave elastography may assist in identifying patients who are more likely to benefit from biopsy, while helping to reduce unnecessary invasive procedures in selected cases.

Overall, shear-wave elastography may support clinical decision-making in selected patients. Further studies are needed to confirm these findings and to define its role in the diagnostic approach to primary Sjögren’s syndrome.

## Figures and Tables

**Table 1:** Baseline characteristics of the study population.

Variable	Patients (n = 53)	Controls (n = 40)	*p*-Value
Age (years), mean ± SD	49.92 ± 12.48	47.10 ± 13.38	0.29
Female sex, n (%)	53 (100)	40 (100)	—

Abbreviations: SD, standard deviation. Note: Data are presented as mean ± SD or number (%). The *p*-value was calculated using the independent samples *t*-test. The *p*-value was not calculated for sex because all participants were female.

**Table 2:** Comparison of parotid shear-wave elastography velocity values (m/s) between pSS patients and controls.

Variable	pSS (n = 53)	Controls (n = 40)	*p*-Value
Shear-wave velocity, m/s (mean ± SD)	2.32 ± 0.58	1.77 ± 0.16	<0.001
Shear-wave velocity, m/s (median [IQR])	2.25 (1.86–2.70)	1.82 (1.69–1.87)	<0.001

Abbreviations: SD, standard deviation; IQR, interquartile range; pSS, primary Sjögren’s syndrome. Note: Between-group comparisons were performed using the Mann–Whitney U test.

**Table 3:** Interobserver reliability of parotid shear-wave elastography measurements.

Measurement	ICC
Right parotid gland SWE	0.735
Left parotid gland SWE	0.787

Abbreviations: ICC, intraclass correlation coefficient; SWE, shear-wave elastography. Note: Interobserver agreement was assessed using a two-way random-effects model with absolute agreement. ICC values were interpreted as follows: <0.50 poor, 0.50–0.75 moderate, 0.75–0.90 good, and >0.90 excellent reliability.

**Table 4:** Age-adjusted difference in shear-wave elastography velocity (m/s) between groups.

Variable	Adjusted β	95% CI	*p*-Value
Shear-wave elastography velocity (m/s) (patients vs. controls)	2.141	1.468–2.814	<0.001

Note: Analysis performed using analysis of covariance (ANCOVA) adjusted for age.

**Table 5:** Diagnostic performance of parotid shear-wave elastography velocity (m/s) according to the optimal ROC cut-off value.

Cut-Off Value (m/s)	Sensitivity (%)	Specificity (%)	Youden Index
2.17	69.0	94.1	0.631

Note: The optimal shear-wave elastography velocity cut-off value was determined using the Youden index from the ROC analysis.

**Table 6:** Parsimonious multivariate logistic regression model for predicting histopathological positivity.

Variable	Odds Ratio	95% CI	*p*-Value
Age	1.007	0.944–1.073	0.839
Shear-wave elastography velocity (m/s)	6.106	0.857–43.533	0.071
Anti-SSA positivity	6.637	0.655–67.260	0.109

Outcome: histopathological positivity (focus score ≥ 1). Note: Parsimonious logistic regression model including clinically relevant variables.

## Data Availability

The data presented in this study are available from the corresponding author upon reasonable request.

## Supplementary Materials

The following supporting information can be downloaded at: https://www.mdpi.com/article/10.3390/diagnostics16071095/s1, Table S1: Age-Matched Comparison of Parotid Shear-Wave Elastography Velocity (m/s).

## Abbreviations

ACR	American College of Rheumatology
ANA	Antinuclear Antibody
ANCOVA	Analysis of Covariance
AUC	Area Under the Curve
CI	Confidence Interval
ENA	Extractable Nuclear Antigen
IQR	Interquartile Range
LSGB	Labial (Minor) Salivary Gland Biopsy
pSS	Primary Sjögren’s Syndrome
ROC	Receiver Operating Characteristic
ROI	Region of Interest
SD	Standard Deviation
SWE	Shear-Wave Elastography
USE	Ultrasound Elastography
US	Ultrasonography
