# Supplementary material for: Diagnostic Performance of Parotid Shear-Wave Elastography for Predicting Histopathological Positivity in Patients with Suspected Primary Sjögren’s Syndrome
Source: Diagnostics (Basel). 2026 Apr 5;16(7):1095. doi: 10.3390/diagnostics16071095 (PMC13073103; doi:10.3390/diagnostics16071095)
Supplement: Supplementary file 1 [file diagnostics-16-01095-s001.zip › diagnostics-4217568-supplementary.pdf]

Supplementary Table S1. Age-Matched Comparison of Parotid Shear-Wave Elastography Velocity (m/s)

Variable	Patients (age-matched)	Controls	p-value
SWE velocity (m/s), mean ± SD	2.54 ± 0.78	1.83 ± 0.18	0.021
SWE velocity (m/s), median (IQR)	2.45 (2.00–3.00)	1.80 (1.70–1.95)	

Note: Nearest-neighbor age matching was performed without replacement. Values are presented as mean ± SD and median (IQR). The Mann–Whitney U test was used for comparison.
